# Tourniquet-induced tissue hypoxia characterized by near-infrared spectroscopy during ankle surgery: an observational study

**DOI:** 10.1186/s12871-019-0740-8

**Published:** 2019-05-10

**Authors:** Liang Lin, Gang Li, Jinlei Li, Lingzhong Meng

**Affiliations:** 1 Department of Anesthesiology, The First Affiliated Hospital, Xiamen University, Xiamen, Fujian Province China; 2 Department of Anesthesiology, Peking University Third Hospital, Beijing, China; 3 Department of Anesthesiology, Yale University School of Medicine, 333 Cedar Street, TMP 3, P.O. Box 208051, New Haven, CT 06520 USA

**Keywords:** Tissue oxygenation, Tourniquet, Ischemia, Hypoxia

## Abstract

**Background:**

Pneumatic tourniquet inflation during extremity surgery leads to profound and prolonged tissue ischemia. Its effect on tissue oxygenation is inadequately studied.

**Methods:**

Patients undergoing elective ankle surgery with tourniquet application participated in this observational cohort study. Somatic and cerebral tissue oxygen saturation (SstO_2_ and SctO_2_) were monitored using tissue near-infrared spectroscopy. Oxygenation was monitored distally (SstO_2_-distal) and proximally to the tourniquet, on the contralateral leg, and the forehead (a total of 4 tissue beds). Tissue oxygenation at different time points was compared. The magnitude, duration, and load (product of magnitude and duration) of tissue desaturation during tourniquet inflation were correlated with tissue resaturation and hypersaturation after tourniquet deflation.

**Results:**

Data of 26 patients were analyzed. The tourniquet inflation time was 120 ± 31 mins. Following a rapid desaturation from 77 ± 8% pre-inflation to 38 ± 20% 10 mins post-inflation, SstO_2_-distal slowly and continuously desaturated and reach the nadir (16 ± 11%) toward the end of inflation. After deflation, SstO_2_-distal rapidly resaturated from 16 ± 11% to 91 ± 5% (i.e., hypersaturation); SstO_2_ monitored proximally to the tourniquet and on contralateral leg had significant but small desaturation (~ 2–3%, *p* <  0.001); in contrast, SctO_2_ remained stable. The desaturation load had a significant correlation with resaturation magnitude (*p* <  0.001); while the desaturation duration had a significant correlation with hypersaturation magnitude (*p* = 0.04).

**Conclusions:**

Tissue dys-oxygenation following tourniquet application can be reliably monitored using tissue oximetry. Its outcome significance remains to be determined.

## Background

A pneumatic tourniquet is commonly employed during extremity surgery to reduce blood loss and facilitate the surgeon’s operation (i.e., a bloodless surgical field). It is intriguing when considering that, although the blood flow is completely or near-completely stopped for a prolonged period, the tissue beds distal to the tourniquet are still alive afterward. In theory, the ischemic tissue would become hypoxic, and the hypoxia would become progressively worse, following the interruption of blood flow as long as the tissue continues to consume oxygen albeit maybe at a much slower rate as a result of the adaptive changes or other factors such as anesthesia [1, 2]. It is enlightening if the change in tissue oxygenation following tourniquet inflation is continuously monitored. The modern tissue oximetry based on near-infrared spectroscopy enables non-invasive, bedside and continuous measurement of the hemoglobin oxygen saturation of the mixed arterial, capillary, and venous blood in the tissue bed that is ~ 2–2.5 cm below the probe. Cerebral tissue oxygen saturation (SctO_2_) monitored on the forehead has been used in clinical care for 20+ years in patients having surgery [3] and cardiac arrest [4]; in contrast, the clinical application of somatic tissue oxygen saturation (SstO_2_), monitored at a peripheral location, is relatively new [5, 6]. The goal of this prospective cohort study was to characterize the tissue dys-oxygenation related to tourniquet application during ankle surgery. The secondary objective was to correlate parameters derived during tourniquet inflation with parameters derived following tourniquet deflation to explore the potential direction for future research.

## Methods

This observational analytic cohort study was approved by the Institutional Review Board for clinical investigations at Yale University, New Haven, Connecticut, USA. Consent to participate in the study was obtained from all patients before surgery.

### Patients and anesthesia

The inclusion criteria were: 1) elective ankle surgery for non-diabetic-related injuries, 2) tourniquet application, and 3) American Society of Anesthesiologists (ASA) physical status score ≤ III. The exclusion criteria were: 1) patient refusal, 2) urgent or emergent surgery, 3) age < 18 years, 4) diabetic foot, 5) peripheral vascular disease, 6) skin condition unsuitable for adhesive oximetry probe, 7) pregnancy and 8) existing neuropathy or myopathy.

All patients received ultrasound-guided peripheral nerve blockade using an insulated needle before surgery. Patients were monitored with electrocardiogram, pulse oximetry, and non-invasive blood-pressure, supplemented with 2 l/min oxygen by nasal cannula, and pre-medicated with intravenous 1–2 mg midazolam and 50–100 μg fentanyl. A single-shot popliteal, sciatic, and saphenous nerve block were performed under in-plane technique with a total of 30 ml 0.5% ropivacaine. Upon arriving at the operating room and following anesthesia induction with intravenous lidocaine, fentanyl and propofol administration, either an endotracheal tube or laryngeal mask airway, at the discretion of the anesthesia team, was placed. Anesthesia was maintained using sevoflurane. The tourniquet was placed on the upper leg and inflated to 300 mmHg during surgery in all patients.

### Tissue oxygenation monitoring

Tissue oxygenation was monitored using a tissue oximeter based on near-infrared spectroscopy (NIRS) (FORE-SIGHT Elite, CASMED, Inc., Branford, Connecticut). In essence, NIRS-measured tissue oxygenation is determined by the balanced between oxygen consumption and supply of the tissue bed which is about 2–2.5 cm below the interrogating probe. In this study, four different tissue beds were monitored in each patient: 1) SstO_2_ distal to the tourniquet (SstO_2_-distal) with the probe placed on the back of the lower leg and about 4 fingers below the popliteal crease; 2) SstO_2_ proximal to the tourniquet (SstO_2_-prox) with the probe placed on the front of the upper leg and about 6 fingers below the femoral crease; 3) SstO_2_ on the contralateral leg (SstO_2_-contra) at the same location as SstO_2_-distal; 4) SctO_2_ with the probe placed on the forehead. Monitoring and data recording started before anesthesia induction and stopped at the end of surgery.

### Data recording and analysis

Tissue oxygenation of different tissue beds was simultaneously and continuously recorded into an excel worksheet by a research laptop at a frequency of one new data point every 2 s. The medians of tissue oxygenation within each minute were used in the analysis. The time points of interest were: immediately before tourniquet inflation (T_0_), 5 mins (T_5_), 10 mins (T_10_), 20 mins (T_20_), 30 mins (T_30_), and 60 mins (T_60_) after tourniquet inflation, immediately before tourniquet deflation (T_end_), and 3–5 min after tourniquet deflation (T_post_). The hypoxic load, defined as the product of the magnitude and duration of tissue desaturation, is quantified by the area under the curve (AUC) encircled by the actual tracing and the straight line of the baseline value (T_0_).

### Statistical analysis

As an exploratory observational study, a power analysis was not performed before the study. Data are expressed as mean ± SD. Paired Student's t-test was used when comparing the changes in tissue oxygenation of the same tissue bed. The correlation between the variables before tourniquet deflation (baseline oxygenation (T_0_), maximal hypoxia (T_end_), hypoxic duration, and hypoxic load (AUC)) and the variables after tourniquet deflation (resaturation magnitude (∆T_post-end_), resaturation rate (%/second), and hyperemic response (∆T_post-0_)) was analyzed using Pearson’s correlation coefficient. The *p* value < 0.05 was considered significant. Statistical analyses were performed using SPSS software (ver. 22.0 for Windows; SPSS Inc., Chicago, IL).

## Results

Thirty-one patients participated in this study. Five patients were excluded from the analysis due to incomplete data (*n* = 4) and conversion of ankle surgery to below-knee amputation (*n* = 1). Data of 26 patients were included in the final analysis. The patient’s demographic data and past medical history were summarized in Table 1. All patients had a tourniquet application, with an average duration of 120 ± 31 mins.

**Table 1:** Demographics, tourniquet time and co-morbidities of the study population (*n* = 26)

Variables	Value & count
Age (year)	48 ± 15
Sex = male (n (%))	16 (62%)
Weight (kg)	89 ± 23
Height (cm)	173 ± 10
BMI	30 ± 7
ASA ≥ II (n (%))	19 (73%)
Leg = right (n (%))	22 (85%)
Hypertension (n (%))	8 (31%)
Diabetes mellitus (n (%))	3 (12%)
Peripheral vascular disease (n (%))	1 (6%)
Chronic lung disease (n (%))	6 (23%)
Cardiovascular diseases (n (%))	1 (6%)

*BMI* body mass index, *ASA* American Society of Anesthesiologists

**Fig. 1:**
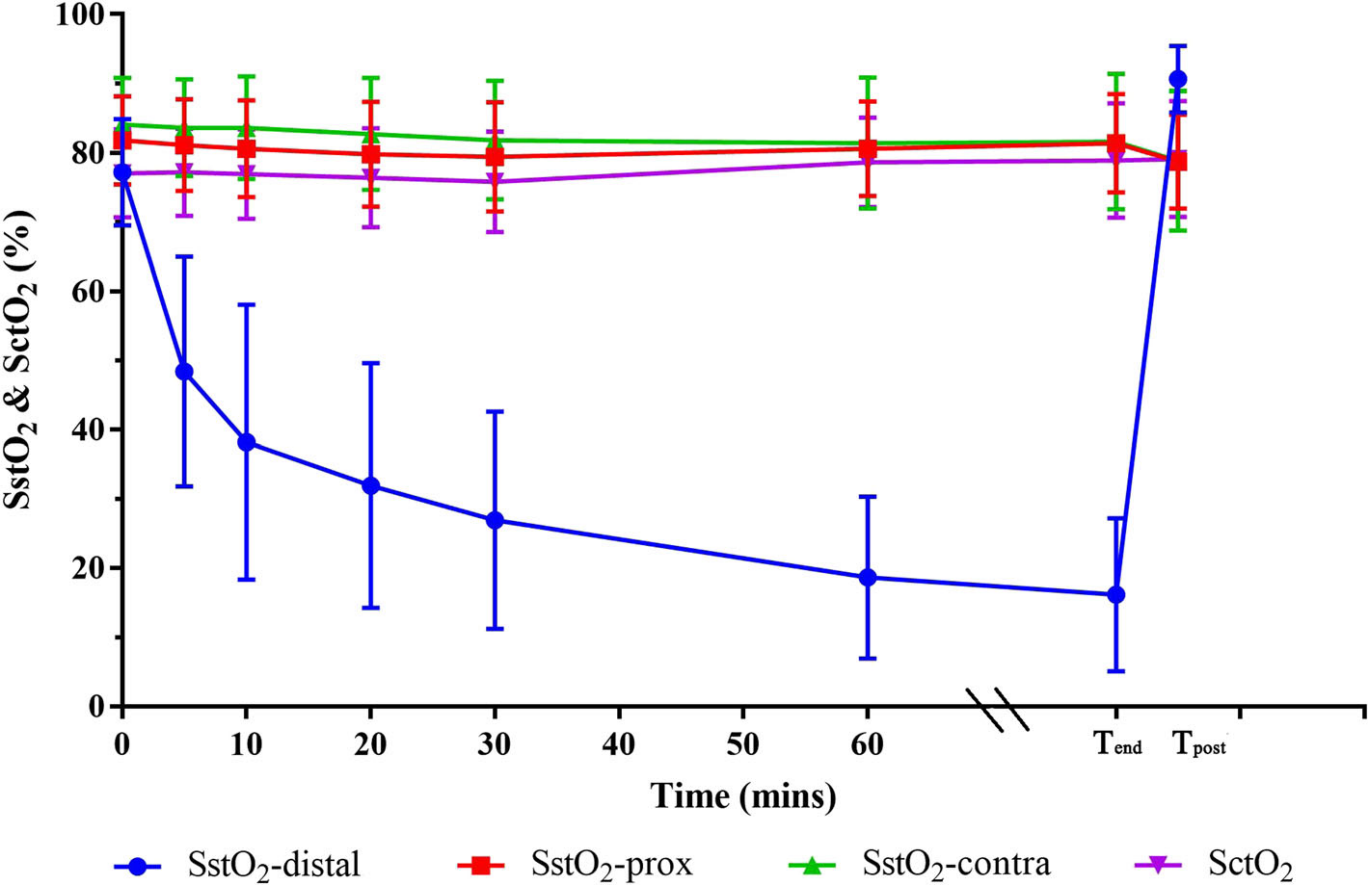
Group mean and standard deviation of somatic tissue oxygen saturation (SstO_2_) monitored distal (SstO_2_-distal) and proximal (SstO_2_-prox) to the tourniquet and on the contralateral leg (SstO_2_-contra) and cerebral tissue oxygen saturation (SctO_2_) monitored on the forehead at different time points. T_end_ = time point at the end of tourniquet inflation; T_post_ = time point 3–5 min after tourniquet deflation

Tissue oxygenation of different tissue beds was summarized in Table 2 and illustrated by Fig. 1. Tourniquet inflation led to a rapid decrease of SstO_2_-distal from 77 ± 8% pre-inflation to 38 ± 20% 10 mins post-inflation (51% relative decrease). SstO_2_-distal slowly, but continuously, trended downward (i.e., desaturation) throughout the rest of the inflation period and did not reach the nadir (16 ± 11, 79% relative decrease) until immediately before the tourniquet deflation. Following tourniquet deflation, there was a rapid increase (469% relative increase) of SstO_2_-distal from the nadir of 16 ± 11% to the peak of 91 ± 5% about 3–5 min post-deflation (i.e., resaturation). The difference between the post-deflation peak value (T_post_) and the pre-inflation baseline value (T_0_) of SstO_2_-distal was 14 ± 8% (18% relative increase) (i.e., hyperemia) (Table 3).

**Table 2:** Absolute values and changes of somatic tissue oxygen saturation (SstO_2_) and cerebral tissue oxygen saturation (SctO_2_) at different time points (*n* = 26)

	T_0_	T_end_	T_post_	ΔT_post-0_^a^	ΔT_post-end_^b^
SstO_2_-distal (%)	77.5 ± 7.7	15.6 ± 9.7	90.9 ± 4.0	13.8 ± 8.0^*^	75.4 ± 11.5^*^
SstO_2_-prox (%)	82.3 ± 6.1	81.1 ± 7.8	78.4 ± 7.3	−3.6 ± 4.5^*^	−2.7 ± 2.3^*^
SstO_2_-contra (%)	81.2 ± 6.8	81.3 ± 10.0	78.3 ± 10.5	−5.6 ± 6.3^*^	− 3.0 ± 3.2^*^
SctO_2_ (%)	77.5 ± 6.2	79.9 ± 7.9	80.1 ± 8.0	2.0 ± 6.3	0.2 ± 2.7

SstO_2_-distal = SstO_2_ distal to the tourniquet; SstO_2_-prox = SstO_2_ proximal to the tourniquet; SstO_2_-contra = SstO_2_ on the contralateral leg; T_0_ = immediately before tourniquet insufflation; T_end_ = immediately before tourniquet deflation; T_post_ = 3–5 min after tourniquet deflation

^*^P < 0.001

^a^paired Student's t-test between T_post_ and T_0_

^b^paired Student's t-test between T_post_ and T_end_

**Table 3:** Association of representative variables of tissue oxygenation with variables of resaturation and hyperemia following tourniquet deflation (*n* = 26)

Variable	Resaturation magnitude (∆T_post-end_)		Resaturation rate (%/second)		Hyperemic response (∆T_post-0_)	
	*R* value	*P* value	*R* value	*P* value	*R* value	*P* value
Baseline oxygenation (T_0_)	0.02	0.92	−0.01	0.95	−0.87	< 0.001
Maximal hypoxia (T_end_)	−0.94	< 0.001	0.43	0.03	−0.19	0.34
Hypoxic duration	0.26	0.20	−0.17	0.41	0.41	0.04
Hypoxic load (AUC)	0.66	< 0.001	−0.08	0.70	0.09	0.68

T_0_ = immediately before tourniquet insufflation; T_end_ = immediately before tourniquet deflation; T_post_ = 3–5 min after tourniquet deflation. ∆T_post-end_ = difference between tissue oxygenation immediately before and after tourniquet deflation; ∆T_post-0_ = difference between tissue oxygenation immediately after tourniquet deflation and before tourniquet inflation (baseline); AUC = area under curve

**Fig. 2:**
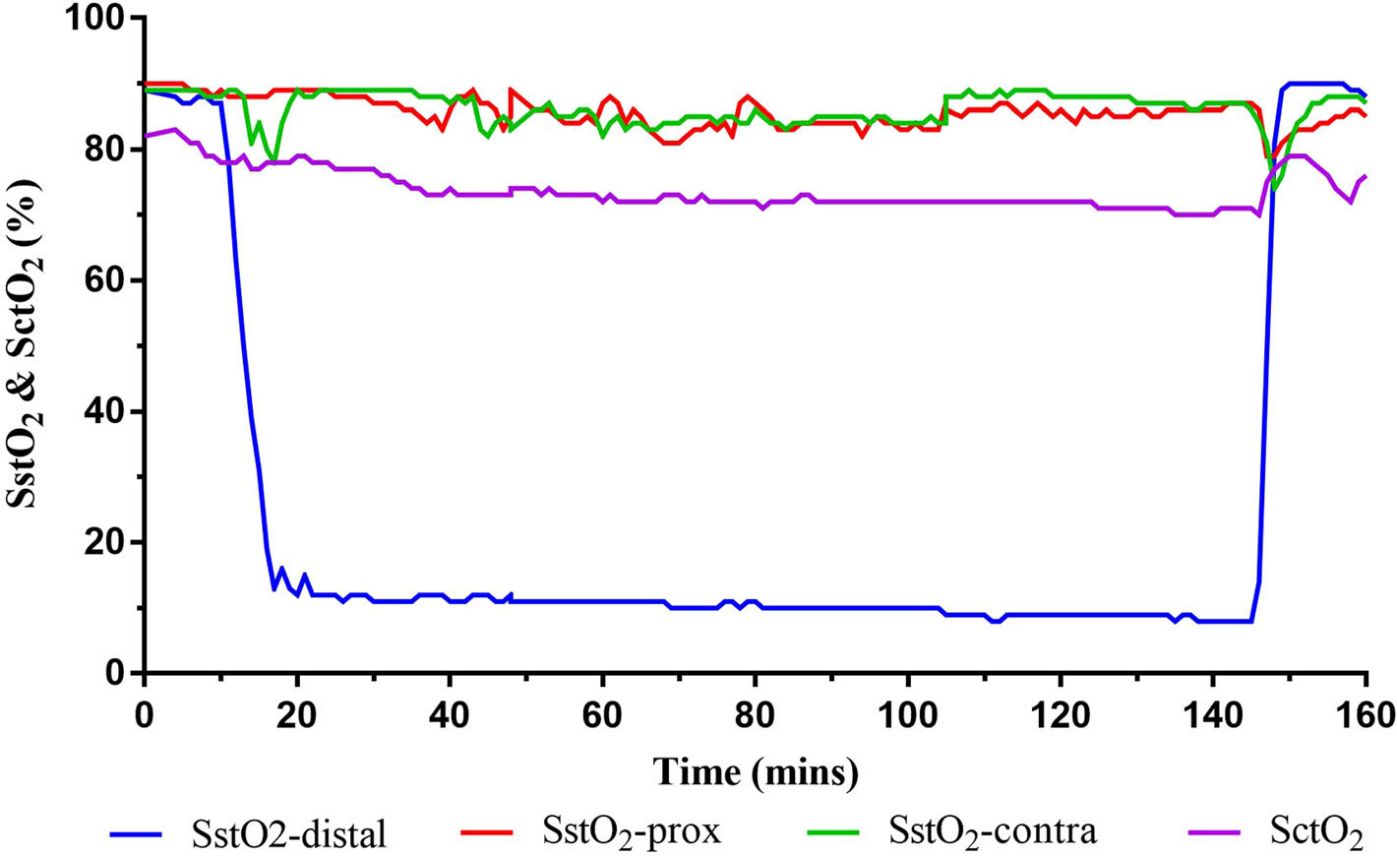
Real-time tracing of somatic tissue oxygen saturation (SstO_2_) monitored distal (SstO_2_-distal) and proximal (SstO_2_-prox) to the tourniquet and on the contralateral leg (SstO_2_-contra) and cerebral tissue oxygen saturation (SctO_2_) monitored on the forehead in a 21-year old college student

The oxygenation of other tissue beds, including SstO_2_-prox, SstO_2_-contra and SctO_2_, remained stable throughout the ischemic period from T_0_ to T_end_. The tourniquet deflation led to a relative decrease of both SstO_2_-prox and SstO_2_-contra of 3–4% (*p* <  0.05); in contrast, SctO_2_ remained relatively stable following tourniquet deflation, albeit it had a small increase in the 21-year old patient illustrated in Fig. 2.

The resaturation magnitude (∆T_post-end_) had a significant correlation with maximal hypoxia (T_end_) (*p* <  0.001) and hypoxic load (AUC) (*p* <  0.001). The resaturation rate (%/second) had a significant correlation with maximal hypoxia (T_end_) (*p* = 0.03). The hyperemic response (∆T_post-0_) had a significant correlation with both baseline oxygenation (T_0_) (*p* <  0.001) and hypoxic duration (*p* = 0.04).

## Discussion

This study showed that extreme tissue hypoxia incurred by tourniquet application can be reliably and continuously measured using NIRS-based tissue oximetry. The hypoxic load (AUC) is significantly associated with the magnitude of the reperfusion-related resaturation (∆T_post-end_), but not the rebound hyperemia (∆T_post-0_). In comparison, the ischemic time is significantly associated with the rebound hyperemia (∆T_post-0_), but not the magnitude of resaturation (∆T_post-end_). The magnitudes, durations, and loads of tissue hypoxia during tourniquet inflation vary among different patients; however, the clinical significance of these parameters remains to be determined.

In 1904, Harvey Cushing first described the clinical application of pneumatic tourniquet [7]. Tourniquet is currently widely used during upper and lower extremity surgery to facilitate the surgeon’s operation by rendering a bloodless surgical field. It is a milestone event in medical history. However, tourniquet is not risk-free. Various post-tourniquet complications have been reported such as nerve palsy [8], vascular injuries [9], wound hypoxia [10], abnormal electromyography and muscle weakness [11]. If given enough time, the tissues that are distal to the tourniquet will eventually die. However, the time limit of safe tourniquet inflation during extremity surgery remains controversial [12–14]. The dogma of 90 mins is based on animal studies [15, 16]. In patients undergoing knee surgery, Gidlöf et al. showed that tourniquet-induced prolonged ischemia (90–180 min) led to a progressively worsening endothelial injury [17]. Many other studies showed that tourniquet-induced extreme ischemia (> 4 h) can lead to irreversible skeletal muscle injury [18, 19].

The effect of tourniquet inflation on NIRS-measured tissue oxygenation in humans has been previously reported [20]. However, the tissue beds monitored and the research aims in these studies are different from our study. The study performed by Song et al. monitored cerebral, not somatic, tissue bed in patients undergoing total knee replacement surgery [21]. Tujjar et al. only monitored the tissue bed that was distal to the tourniquet in patients undergoing upper extremity surgery [22]. In healthy volunteers, Muellner et al. studied the effects of different tourniquet inflation pressures on tissue oxygenation based on the monitoring of the tissue bed distal to the tourniquet only [23]. In patients undergoing ankle fracture repair, Shadgan et al. studied the relationship between tissue oxygenation and oxidative muscle injury based on the monitoring of the tissue beds distal to the tourniquet and on the contralateral leg [24].

The reactive hyperemia following tourniquet release is a well-documented phenomenon [25]. De Backer and Durand advocated the use of reactive hyperemia as an indicator of the microvascular reserve [26], as corroborated by the observation that the magnitude of reactive hyperemia is reduced in septic patients compared with control subjects [27]. In a rat model, Kim et al. showed that NIRS-measured tissue oxygenation had an overshoot (i.e., higher than baseline) following a 2-h, not 3-h tourniquet inflation, suggesting an association between the duration of ischemia and the magnitude of hyperemic response [28].

The *severity* of tourniquet-induced ischemia is traditionally gauged by the duration of tourniquet inflation. However, this approach may have overlooked the dynamic nature of tissue ischemia in an individual patient and the variability of ischemic severity among different patients, as suggested by both our study and the previous studies [21–24]. Moreover, the consequence of tissue ischemia is determined not only by the ischemic duration, but also the metabolic demand as suggested by the association between slow energy consumption and delayed ultrastructural damage in the canine ischemic model [29]. Tissue oximetry, which measures the balance between tissue oxygen consumption and supply continuously and non-invasively, is a promising technology in assessing the *severity* of tourniquet-induced ischemia in individual patients.

Skeletal muscle can rapidly adjust its energy expenditure and production during acute ischemia [30, 31]. The ATPs reserved in muscle fibers only last for a few seconds [32]. However, the skeletal muscle can remarkably replenish energy via two distinctive anaerobic pathways. The pathway of anaerobic glycolysis can sustain muscle activity for a few minutes [33]; while the pathway of phosphocreatine degradation can sustain muscle activity from minutes to hours [34]. As a result, the ATPs in skeletal muscle fall at a very low rate during the first 3–4 h of ischemia [35, 36]. However, tissue damage characterized by cell necrosis and apoptosis eventually ensues about 6–7 h after the onset of ischemia when the glycogen and phosphocreatine reserves are exhausted [37].

An interesting observation of our study is the rapid desaturation for about 10 mins followed by a slow but continuous desaturation for the remaining ischemic period in the tissue bed distal to tourniquet. This phenomenon may be secondary to the adaptive adjustment of metabolic activity made by primarily muscular tissue. Although SctO_2_ remained stable following tourniquet deflation based on the average of all patients, the 21-year-old physically fit college student had a notable increase in SctO_2_, a change different to most other patients (Fig. 2). It may relate to the metabolites (including carbon dioxide) generated by the ischemic tissue which were flushed into cerebral circulation and led to cerebral vasodilation following tourniquet deflation. This 21-year-old young patient may have a more robust cerebral vasoreactivity to carbon dioxide than older patients (the average age of all patients = 48 years). Nonetheless, the exact cause and the clinical significance of this outlier remain to be elucidated.

This study did not evaluate the complications associated tourniquet application and thus cannot tell the relationship between tissue NIRS parameters and ischemia-related outcomes. This is a major limitation of our study. Also, all patients in our study had a peripheral nerve block, which makes it difficult to extrapolate the findings of this study in patients without nerve block. We found a considerable variation in both the rate and magnitude of tissue desaturation following tourniquet inflation. One of the potential causes of this inter-individual variation may relate to the thickness of the skin and subcutaneous tissue because thick superficial layers may preclude the near-infrared light from interrogating the deeper muscular tissue.

## Conclusion

NIRS-based tissue oximetry can reliably and continuously measure tissue desaturation, resaturation and hypersaturation during tourniquet application. The desaturation load is associated the magnitude of resaturation; while the desaturation duration is associate with the magnitude of hypersaturation. The clinical value of tissue oximetry in patients receiving tourniquet application needs to be determined by future research.

## Abbreviations

∆T_post-0_: T_post_ – T_0_
∆T_post-end_: T_post_ – T_end_
AUC: Area under the curve
SctO_2_: Cerebral tissue oxygen saturation
SstO_2_: Somatic tissue oxygen saturation
SstO_2_-contra: SstO_2_ on the contralateral leg
SstO_2_-distal: SstO_2_ distal to the tourniquet
SstO_2_-prox: SstO_2_ proximal to the tourniquet
T_0_: Immediately before tourniquet insufflation
T_end_: Immediately before tourniquet deflation
T_post_: 3–5 min after tourniquet deflation
